# Emergency Department Visits, Care, and Outcome After Stroke and Myocardial Infarction During the COVID-19 Pandemic Phases

**DOI:** 10.1016/j.cjco.2021.06.002

**Published:** 2021-10-27

**Authors:** Amy Y.X. Yu, Douglas S. Lee, Manav V. Vyas, Joan Porter, Mohammed Rashid, Jiming Fang, Peter C. Austin, Michael D. Hill, Moira K. Kapral

**Affiliations:** a Department of Medicine (Neurology), University of Toronto, Sunnybrook Health Sciences Centre, Toronto, Ontario, Canada; b ICES, Toronto, Ontario, Canada; c Department of Medicine (Cardiology), University of Toronto–University Health Network, Toronto, Ontario, Canada; d Department of Clinical Neurosciences, Community Health Sciences, and Hotchkiss Brain Institute, University of Calgary, Calgary, Alberta, Canada; e Department of Medicine (General Internal Medicine), University of Toronto–University Health Network, Toronto, Ontario, Canada

## Abstract

**Background:**

It is not known if initial reductions in hospitalization for stroke and myocardial infarction early during the coronavirus disease–2019 pandemic were followed by subsequent increases. We describe the rates of emergency department visits for stroke and myocardial infarction through the pandemic phases.

**Methods:**

We used linked administrative data to compare the weekly age- and sex-standardized rates of visits for stroke and myocardial infarction in Ontario, Canada in the first 9 months of 2020 to the mean baseline rates (2015-2019) using rate ratios (RRs) and 95% confidence intervals (CIs). We compared care and outcomes by pandemic phases (pre-pandemic was January-March, lockdown was March-May, early reopening was May-July, and late reopening was July-September).

**Results:**

We identified 15,682 visits in 2020 for ischemic stroke (59.2%; n = 9279), intracerebral hemorrhage (12.2%; n = 1912), or myocardial infarction (28.6%; n = 4491). The weekly rates for stroke visits in 2020 were lower during the lockdown and early reopening than at baseline (RR 0.76, 95% CI [0.66, 0.87] for the largest weekly decrease). The weekly rates for myocardial infarction visits were lower during the lockdown only (RR 0.61, 95% CI [0.46, 0.77] for the largest weekly decrease), and there was a compensatory increase in visits following reopening. Ischemic stroke 30-day mortality was increased during the lockdown phase (11.5% pre-coronavirus disease; 12.2% during lockdown; 9.2% during early reopening; and 10.6% during late reopening, *P* = 0.015).

**Conclusion:**

After an initial reduction in visits for stroke and myocardial infarction, there was a compensatory increase in visits for myocardial infarction. The death rate after ischemic stroke was higher during the lockdown than in other phases.

## Résumé

**Contexte:**

Nous ignorons si les réductions des taux d'hospitalisations pour un AVC et un infarctus du myocarde observées au début de la pandémie de coronavirus de 2019 ont été suivies d'une hausse de ces taux. Nous décrivons ici les taux de visites aux services des urgences pour un AVC et un infarctus du myocarde pendant toutes les phases de la pandémie.

**Méthodes:**

À partir de données administratives couplées, nous comparons les taux hebdomadaires de visites pour un AVC et un infarctus du myocarde, normalisés en fonction de l’âge et du sexe, en Ontario, au Canada, effectuées pendant les neuf premiers mois de 2020, avec les taux moyens de base (2015-2019), en utilisant des ratios des taux (RR) et des intervalles de confiance (IC) à 95 %. Nous avons comparé les soins et les issues selon les phases de la pandémie (avant la pandémie : de janvier à mars; confinement : de mars à mai; début de la réouverture : de mai à juillet; phase tardive de la réouverture : de juillet à septembre).

**Résultats:**

Nous avons répertorié 15 682 visites effectuées en 2020 pour un AVC ischémique (59,2 %; n = 9 279), une hémorragie intracérébrale (12,2 %; n = 1 912) ou un infarctus du myocarde (28,6 %; n = 4 491). Les taux hebdomadaires de visites pour un AVC effectuées en 2020 étaient inférieurs pendant la période de confinement et le début de la réouverture comparativement à la période de base (RR : 0,76; IC à 95 % : 0,66-0,87 pour la plus grande baisse hebdomadaire). Les taux hebdomadaires de visites pour un infarctus du myocarde étaient inférieurs pendant la période de confinement seulement (RR : 0,61; IC à 95 % : 0,46-0,77 pour la baisse hebdomadaire la plus importante); une hausse compensatrice du nombre de visites a été notée après la réouverture. Le taux de mortalité à 30 jours des suites d'un AVC ischémique était plus élevé pendant la période de confinement (11,5 % avant la pandémie de coronavirus; 12,2 % pendant le confinement; 9,2 % au début de la période de réouverture; 10,6 % pendant la phase tardive de la réouverture, *p* = 0,015).

**Conclusion:**

Après une réduction initiale du nombre de visites motivées par un AVC et un infarctus du myocarde, on a noté une hausse compensatrice du nombre de visites motivées par un infarctus du myocarde. Le taux de mortalité des suites d'un AVC ischémique était plus élevé pendant la période de confinement que pendant les autres périodes.

Several reports have shown reductions in hospitalization for stroke and myocardial infarction following the World Health Organization announcement of the Coronavirus disease 2019 (COVID-19) global pandemic on March 11, 2020.^1^, ^2^, ^3^, ^4^ In the early phases of the pandemic, when less was understood about the virus, and the population prevalence of COVID-19 in Canada was still relatively low, a change in patient health-seeking behaviouris expected to have played a prominent role in the decrease in hospitalizations, rather than there having been a true reduction in events.

Published data were mainly from the initial phase of the pandemic, between March and May 2020, and less is understood about the effects of the pandemic on hospital visits, processes of care, and clinical outcomes after a cardiovascular or cerebrovascular event, beyond the early period. It is not known if the volume of hospital visits has remained low, has normalized, or has had a compensatory increase after the initial pandemic phase, but a better understanding of the collateral effects of the pandemic on care and outcomes will inform health services planning. Furthermore, although several studies have reported reductions in acute revascularization procedures for stroke and cardiac events,^3^^,^^5^^,^^6^ it is not known if these reductions are proportional to the decrease in hospital visits.

Our objective was to assess the changes in emergency department (ED) visit volume, care processes, and outcomes for stroke and myocardial infarction from before compared with after the declaration of the pandemic, and through the various phases of restrictions, in the population of Ontario, Canada.

## Methods

### Study setting and cohort

The study cohort consisted of all community-dwelling adults residing in Ontario, which is Canada's most populous province (14 million residents), and where the first confirmed Canadian COVID-19 case was reported.^7^ Ontario has a universal healthcare system, with nearly all (> 99%) residents of Ontario insured.

### Pandemic timeline in Ontario

The phases of the pandemic were defined using the restrictions imposed by the Ontario government.^8^ The pre-pandemic phase was from January 1 to March 10, 2020 (weeks 1-10). The lockdown phase was from March 11 to May 19, 2020 (weeks 11-20), when a provincial state of emergency was declared, in-person schools were moved online, and nonessential businesses were closed. The early reopening phase was from May 20 to July 14, 2020 (weeks 21-28), when all regions in Ontario entered the first phase of reopening in which nonessential businesses were allowed to open according to regional public health guidelines, and gatherings were permitted for < 10 people indoors and < 50 people outdoors. Finally, the late reopening phase was from July 15 to September 30, 2020 (weeks 29-40), when the provincial state of emergency ended, restaurants allowed indoor dining at reduced capacity, gatherings were permitted for < 50 people indoors and < 100 people outdoors, and in-person schools resumed. September 2020 also marked the start of the second wave in Ontario, but the next province-wide lockdown was not until December 2020.

### Primary outcome

The primary outcome was any ED visit with a main diagnosis of stroke (International Classification of Diseases, 10th revision, codes H34.1, I63.x, I64.x for ischemic stroke, and I61.x, I60.x for intracerebral hemorrhage and nontraumatic subarachnoid hemorrhage)^9^ or myocardial infarction (I21.x, I22.x)^10^^,^^11^ between January and September from 2015 to 2020. These data were obtained from the National Ambulatory Care Reporting System database, which includes data on all ED encounters in Ontario.

### Secondary outcomes

The secondary outcomes of interest were the processes of care, and outcomes in patients evaluated in 2020, during the various phases of the COVID-19 pandemic. Processes-of-care metrics included the proportion of patients in the following scenarios: arriving by ambulance; admitted to hospital from the ED; evaluated at a comprehensive regional stroke centre (a designated tertiary-care hospital that serves patients with acute stroke who require advanced stroke care or expertise)^12^; treated with intravenous thrombolysis^13^ or endovascular thrombectomy^14^ among the patients with ischemic stroke; and managed with coronary angiogram, percutaneous coronary intervention, or coronary artery bypass grafting surgery^15^ among patients with myocardial infarction. Outcomes included death within 30 days from the ED visit and, for patients who were admitted to the hospital, the proportion discharged to home. We described patient characteristics, including age, sex, neighbourhood income quintile, and rurality, defined as small town (population of < 10,000 people), medium urban (population of 10,000 to 100,000 people), or large urban (population of > 100,000 people), based on the patient's home postal code. We used linked data from administrative databases that have been extensively validated for research purposes to obtain this information (Supplemental Table S1).^16^^,^^17^ Deterministic linkage was performed using unique encoded identifiers and analyzed at ICES (formerly known as the Institute for Clinical Evaluative Sciences), an independent, nonprofit research institute.

### Statistical methods

The age- and sex-standardized weekly rates of ED visits for stroke or myocardial infarction from January to September 2020 were compared to the baseline rates calculated by combining the corresponding weekly visit standardized rates from each year from 2015 to 2019. The 2015 adult population of Ontario was used as the reference population. The 95% confidence interval (CI) around weekly rates was calculated based on the Poisson distribution. We then calculated the rate ratio (RR) comparing each week in 2020 to the corresponding week in the baseline period (2015-2019) and derived a 95% CI using 1000 bootstrap samples. Given that some cases of myocardial infarction initially may be diagnosed as unstable angina in the ED, we performed a sensitivity analysis, including visits with a diagnosis of angina (I20.x) to further understand visits for acute cardiac events.^18^ For additional context, we calculated the standardized rates of COVID-19 cases in Ontario obtained from the Ontario Laboratories Information System. Patient characteristics, processes of care, and outcomes by the different phases of the pandemic for visits that occurred in 2020 were compared using the χ^2^ test for categorical variables, and the Kruskal–Wallis test for the median of continuous variables. All data analyses were performed using SAS Enterprise Guide version 7.1 (SAS Institute Inc., Cary, NC).

### Research ethics

The use of data in this project was authorized under section 45 of Ontario's Personal Health Information Protection Act, without the requirement for research ethics board approval.

## Results

**Figure 1:**
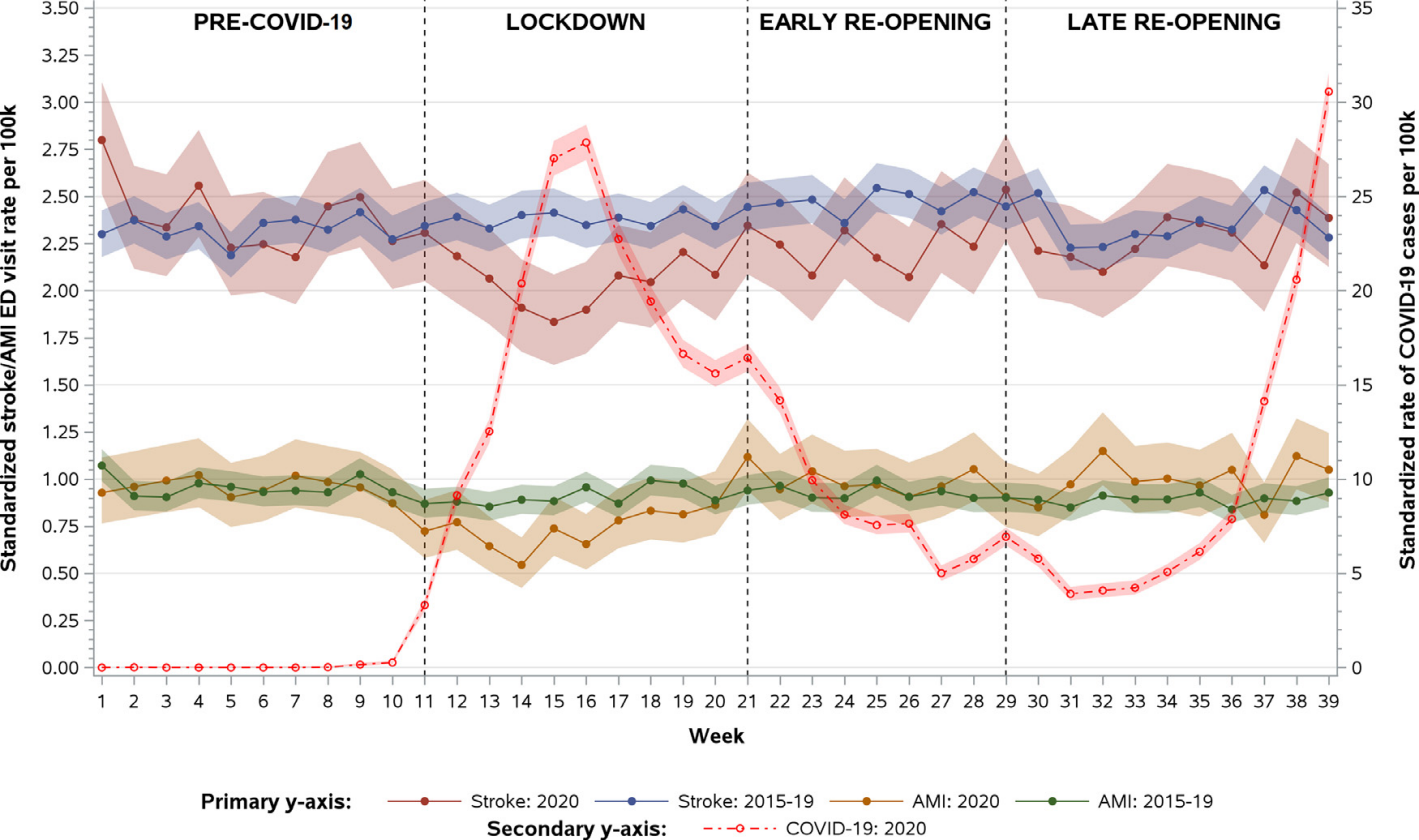
Standardized rates of emergency department (ED) visits for stroke and acute myocardial infarction (AMI) events per 100,000 people, with 95% confidence intervals, comparing 2020 to baseline (2015-2019).

**Figure 2:**
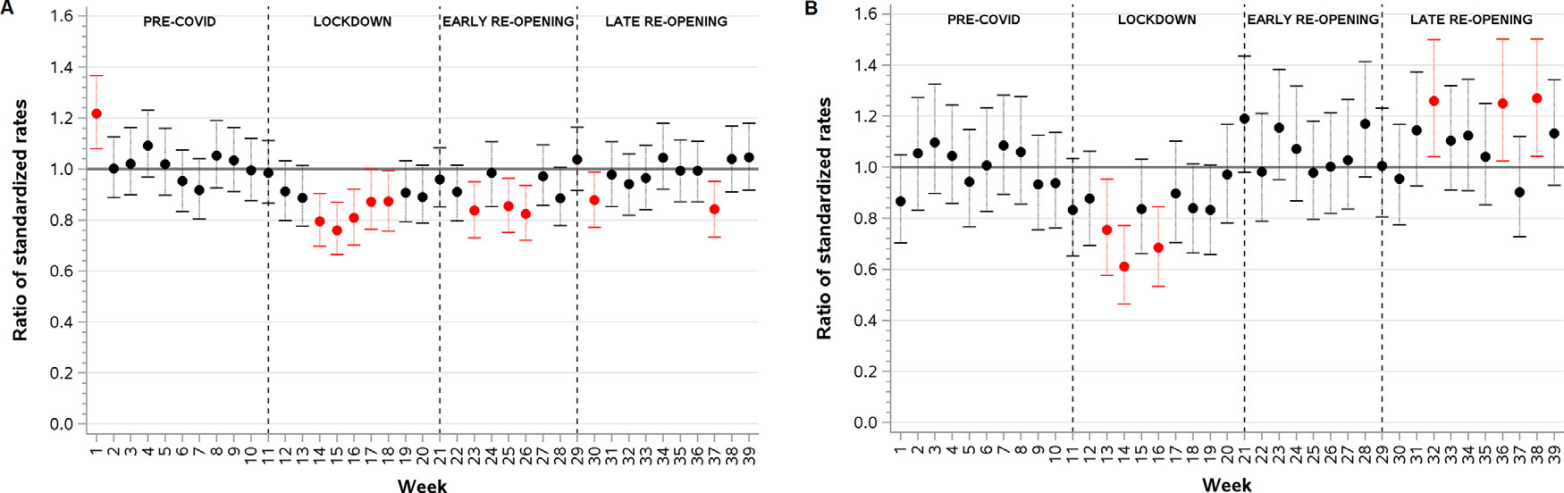
Rate ratios and 95% confidence intervals comparing standardized rate of emergency department visits in 2020 to baseline (2015-2019) for (**A**) stroke and (**B**) myocardial infarction. Rate ratios and 95% confidence intervals in **red** indicate a statistically significant difference in the 2020 visit rate compared to that in the baseline period.

Between January 1 and September 30, 2020, there were 15,682 visits to the ED for ischemic stroke (59.2%; n = 9279), intracerebral hemorrhage (12.2%; n = 1912), and myocardial infarction (28.6%; n = 4491) in 15,323 unique patients. Compared to baseline rates in the preceding 5 years, the weekly visit rates for stroke in 2020 were lower during the lockdown phase (Fig. 1). The largest difference was in the week of April 8 (week 15: 1.83 per 100,000 in 2020 compared to 2.41 in 2015-2019; RR 0.76, 95% CI [0.66, 0.87]; Fig. 2A). In the early reopening phase, rates were still lower than baseline rates for several weeks, and there was no compensatory increase in rates in the late reopening phase.

**Figure 3:**
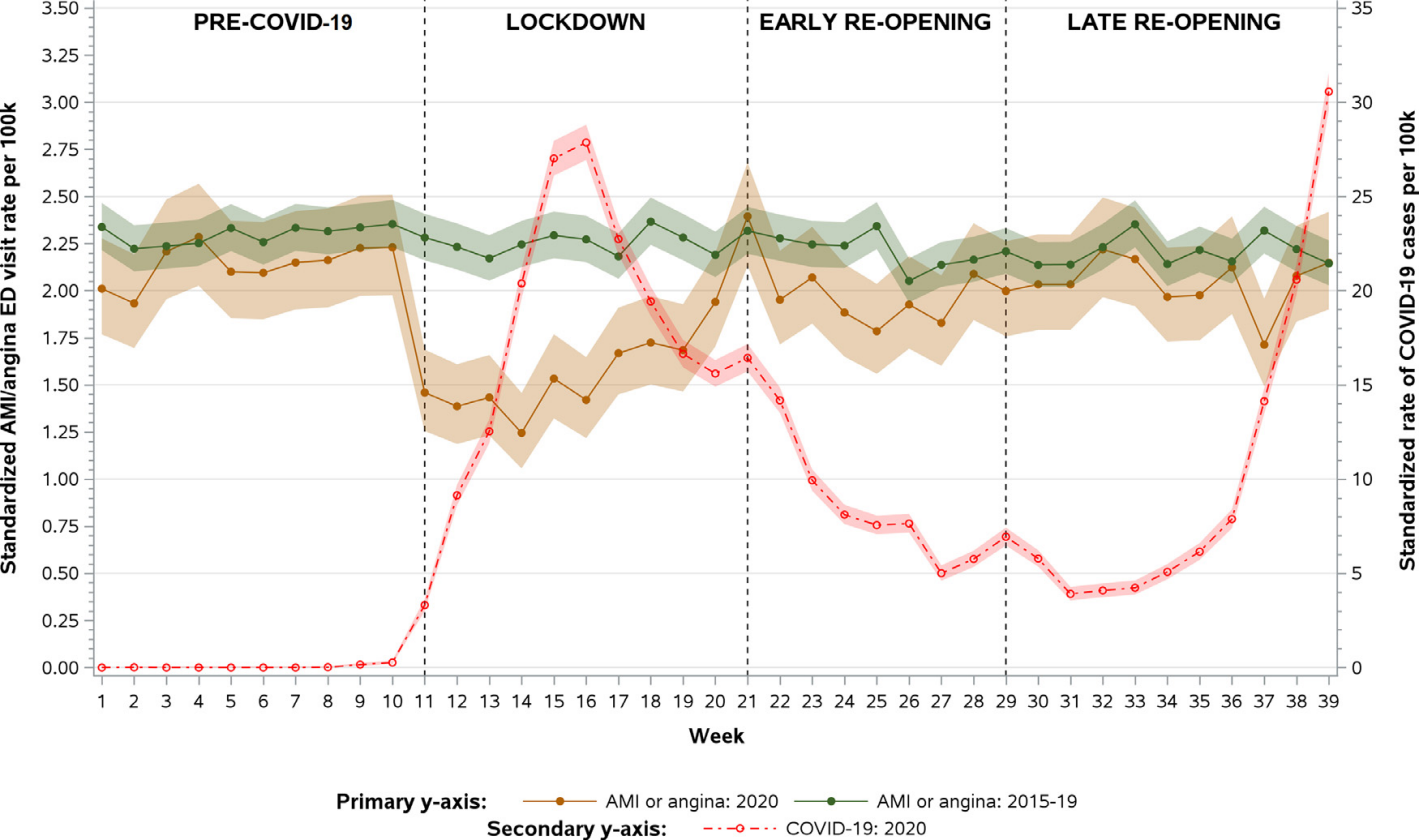
Standardized rates of emergency department (ED) visits for acute myocardial infarction (AMI) or angina per 100,000 people, with 95% confidence intervals, comparing 2020 to baseline (2015-2019).

The weekly visit rates for myocardial infarction were reduced for only 3 weeks during the lockdown phase (Fig. 1). The largest difference was in the week of April 1 (week 14: 0.54 per 100,000 in 2020, compared to 0.89 in 2015-2019; RR 0.61, 95% CI [0.46, 0.77]; Fig. 2B). However, visit rates in 2020 were higher than baseline rates in 3 of the 7 weeks in the late reopening phase. In a sensitivity analysis, we identified an additional 78,038 visits for angina between January and September 2020. The drop in visit volume with a diagnosis of angina and myocardial infarction during the lockdown phase was greater than the reduction seen for myocardial infarction only (Fig. 3), but the rates still quickly recovered to baseline in the early reopening phase, without a compensatory increase. Finally, to better illustrate the 5-year temporal trends, we showed visit rates for each individual year, in Supplemental Figure S1.

In the first 9 months of 2020, the weekly rates of COVID-19 cases peaked in the first wave around week 15, which coincided with the lowest rates of ED visits for stroke and myocardial infarction (Fig. 1). September 2020 marked the start of the second COVID-19 wave in Ontario, but there was no reduction in visit rates despite the volume of COVID-19 cases being comparable to that at the peak of the first wave.

Baseline patient characteristics of the patients who presented in 2020 were similar in the 4 different phases of the pandemic, except for rurality of home location, for which there was a decrease in the number of ED visits by patients living in large urban areas, relative to those living in small towns, during the lockdown and early reopening phases compared to the pre-pandemic and late reopening phases (Table 1). Processes of care are presented by pandemic phase for ischemic stroke, intracerebral hemorrhage, and myocardial infarction separately (Table 2). During the lockdown phase, patients were more likely to arrive by ambulance, compared to the other phases, for all 3 conditions.

**Table 1:** Characteristics of patients with emergency department visits for stroke and myocardial infarction between January and September 2020

Characteristic	Pre-COVID-19: weeks 1–10	Lockdown: weeks 11–20	Early reopening: weeks 21–28	Late reopening: weeks 29–40	*P*
	n = 4232	n = 3544	n = 3278	n = 4628	
Median age, y (Q1, Q3)	72 (62–82)	72 (61–82)	71 (61–81)	72 (62–82)	0.123
Female	1835 (43.4)	1559 (44.0)	1446 (44.1)	1995 (43.1)	0.771
Neighbourhood income quintile					0.537
1 (lowest income)	1016 (24.0)	810 (22.9)	743 (22.7)	1065 (23.0)	
2	875 (20.7)	807 (22.8)	699 (21.3)	991 (21.5)	
3	866 (20.5)	710 (20.0)	695 (21.2)	920 (19.9)	
4	745 (17.6)	605 (17.1)	564 (17.2)	856 (18.5)	
5 (highest income)	730 (17.2)	612 (17.3)	577 (17.6)	796 (17.2)	
Rurality					0.012
Large urban	3045 (72.0)	2522 (71.2)	2275 (69.4)	3382 (73.1)	
Medium urban	532 (12.6)	458 (12.9)	420 (12.8)	533 (11.5)	
Small town	655 (15.5)	564 (15.9)	583 (17.8)	713 (15.4)	
Diagnosis					0.002
Ischemic stroke	2515 (59.4)	2159 (60.9)	1907 (58.2)	2698 (58.3)	
Intracerebral hemorrhage	519 (12.3)	463 (13.1)	372 (11.3)	558 (12.1)	
Myocardial infarction	1198 (28.3)	922 (26.0)	999 (30.5)	1372 (29.6)	

Values are n (%), unless otherwise indicated. Weeks 1-10 are January 1 to March 10, 2020. Weeks 11-20 are March 11 to May 19, 2020. Weeks 21-28 are May 20 to July 14, 2020. Weeks 29-40 are July 15 to September 30, 2020.

Q, quintile.

**Table 2:** Processes of acute care by coronavirus disease 2019 (COVID-19) pandemic phases

Process of acute care	Pre-COVID; weeks 1–10	Lockdown; weeks 11–20	Early reopening; weeks 21–28	Late reopening; weeks 29–40	*P*
Ischemic stroke	n = 2515	n = 2159	n = 1907	n = 2698	

Arrival by ambulance	1598 (63.5)	1521 (70.4)	1213 (63.6)	1770 (65.6)	< 0.001
Comprehensive regional stroke centre care	987 (39.2)	845 (39.1)	763 (40.0)	1102 (40.8)	0.578
Intravenous thrombolysis	348 (13.8)	308 (14.3)	250 (13.1)	341 (12.6)	0.351
Endovascular thrombectomy	174 (6.9)	147 (6.8)	114 (6.0)	181 (6.7)	0.619

Intracerebral hemorrhage	n = 519	n = 463	n = 372	n = 558	

Arrival by ambulance	383 (73.8)	375 (81.0)	275 (73.9)	423 (75.8)	0.035
Comprehensive regional stroke centre care	279 (53.8)	267 (57.7)	199 (53.5)	310 (55.6)	0.564

Myocardial infarction	n = 1198	n = 922	n = 999	n = 1372	

Arrival by ambulance	613 (51.2)	523 (56.7)	479 (47.9)	691 (50.4)	0.001
Coronary angiogram	791 (66.0)	629 (68.2)	714 (71.5)	966 (70.4)	0.025
Percutaneous coronary intervention	523 (43.7)	429 (46.5)	482 (48.2)	633 (46.1)	0.187
Coronary artery bypass grafting surgery	103 (8.6)	73 (7.9)	94 (9.4)	114 (8.3)	0.678

Values are n (%), unless otherwise indicated. Weeks 1-10 are January 1 to March 10, 2020. Weeks 11-20 are March 11 to May 19, 2020. Weeks 21-28 are May 20 to July 14, 2020. Weeks 29-40 are July 15 to September 30, 2020.

Among patients with ischemic stroke, the proportion of patients treated with intravenous thrombolysis, treated with endovascular thrombectomy, or assessed at a comprehensive stroke centre was stable throughout the pandemic phases. Similarly, for those with myocardial infarction, the proportion of patients undergoing coronary angiogram or revascularization procedures was largely stable throughout the pandemic, but the absolute number of procedures was reduced. Death within 30 days was stable throughout the pandemic after intracerebral hemorrhage and myocardial infarction. However, there was a small, but statistically significant increase in death after ischemic stroke during the lockdown phase: 11.5% pre-COVID, 12.2% during lockdown, 9.2% in the early reopening phase, and 10.6% in the late reopening phase (*P* = 0.015; Table 3).

**Table 3:** Clinical outcomes by coronavirus 2019 (COVID-19) pandemic phases

Outcome	Pre-COVID: weeks 1–10	Lockdown: weeks 11–20	Early reopening: weeks 21–28	Late reopening: weeks 29–40	*P*
Died within 30 days	288 (11.5)	264 (12.2)	176 (9.2)	285 (10.6)	0.015
Admitted to hospital	2159 (85.8)	1845 (85.5)	1623 (85.1)	2285 (84.7)	0.687
Ischemic stroke	n = 2515	n = 2159	n = 1907	n = 2698	
Discharged home after admission	1149/2,159 (53.2)	960/1,845 (52.8)	886/1,623 (54.6)	1215/2,285 (53.2)	0.518

Intracerebral hemorrhage	n = 519	n = 463	n = 372	n = 558	

Died within 30 days	169 (32.6)	152 (32.8)	96 (25.8)	166 (29.7)	0.100
Admitted to hospital	478 (92.1)	427 (92.2)	344 (92.5)	512 (91.8)	0.982
Discharged home after admission	179/478 (37.4)	147/427 (34.4)	140/344 (40.7)	191/512 (37.3)	0.360

Myocardial infarction	n = 1198	n = 922	n = 999	n = 1372	

Died within 30 days	150 (12.5)	124 (13.4)	113 (11.3)	164 (12.0)	0.524
Admitted to hospital	1111 (92.7)	839 (91.0)	931 (93.2)	1277 (93.1)	0.217
Discharged home after admission	919/1111 (82.7)	729/839 (86.9)	803/932 (86.3)	1063/1277 (83.2)	0.018

Values are n (%), unless otherwise indicated. Weeks 1-10 are January 1 to March 10, 2020. Weeks 11-20 are March 11 to May 19, 2020. Weeks 21-28 are May 20 to July 14, 2020. Weeks 29-40 are July 15 to September 30, 2020.

## Discussion

We found a reduction in ED visits for stroke and myocardial infarction during the early phases of the pandemic of a magnitude of 25%-40%, consistent with other reports worldwide.^3^, ^4^, ^5^^,^^19^ Visit rates recovered more rapidly for myocardial infarction than for stroke, and there was a signal for a compensatory increase in visits for myocardial infarction in the late reopening phase compared to baseline. The second COVID-19 wave in Ontario started in September 2020, but there was no second drop in visit volume, suggesting that the initial reduction of visits at the beginning of the pandemic is unlikely to represent a true reduction in events in the population.

The stability of visit volume during the second wave may reflect better access to virtual healthcare,^20^ improved public knowledge about hospital measures to reduce the spread of COVID-19, and public service announcement reminders that stroke and heart disease are medical emergencies. However, health-seeking behaviour may remain altered compared to pre-pandemic times, as patients are presenting later after symptom onset or with more severe illness.^6^^,^^19^^,^^21^ Emerging evidence of the indirect negative health effect of COVID-19, including increased risk of stroke and cardiac events, is concerning.^22^^,^^23^ Beyond the direct effects of the virus, the pandemic has led to behaviour, lifestyle, and health system changes that could have an ongoing influence on the incidence of stroke and myocardial infarction, such as reduction of seasonal influenza cases as a result of widespread masking, increases in stress and mental health issues, reduced physical activity, and changes in resource allocation for non-urgent outpatient tests, potentially delaying important routine vascular risk factor management. Ongoing monitoring of disease incidence, care, and outcome is essential.

The processes of care for stroke and myocardial infarction, including hospitalizations, revascularization procedures, and comprehensive stroke-centre care, were reduced proportionally to the decline in ED visit volume, suggesting that once a patient presented for medical attention, care was not affected by the strains of the pandemic on healthcare resources. The increase in 30-day mortality after ischemic stroke during the lockdown period may partially reflect a selection bias for patients with more severe strokes during the lockdown phase, as others have shown that the reduction in hospitalization was more prominent for minor events.^19^^,^^21^ Excess stroke mortality has been reported elsewhere^24^ and warrants further follow-up.

Our findings provide information on the indirect effects of the pandemic, lockdown measures, and the easing of these measure on care and outcomes after stroke and myocardial infarction, which may inform system planning for subsequent waves of disease. This planning is particularly important given the rise of COVID-19 variants that may be more contagious and therefore increase the risk of health systems being overwhelmed.

An important strength of this study is the use of population-level data, allowing assessment of health services utilization and outcomes in important patient groups, such as those living in urban vs rural communities, and reducing selection bias from oversampling from large university-affiliated hospitals, which could be seen in studies using disease-specific registries or hospital-based cohorts.

There are nevertheless limitations. First, availability of administrative health data in Ontario typically lags by 3 to 6 months after the date of the health encounter. Thus, we cannot yet present data on the effect of the pandemic on stroke and myocardial infarction during the second wave. The pandemic has highlighted the urgent need for real-time access to health data for monitoring of healthcare and outcomes. In addition, although misclassification error is inherent to studies that use administrative data, any misclassification is likely stable throughout the study period, because the Canadian coding standards have not changed. The observed change in diagnosis during the pandemic is unlikely to be explained by misclassification errors. Second, we could not assess the severity of events. The proportion of people arriving by ambulance increased during the peak of the first wave, which could reflect increased event severity or more social isolation during the pandemic. Third, the outcomes of individuals who did not present for symptoms of stroke and myocardial infarction cannot be assessed. The current study focuses on changes in ED visits and subsequent care and outcomes, but further work on overall disease incidence in the context of all-cause excess population mortality is needed.^25^

Our study also excludes residents of long-term care facilities who may have had different needs and access to care during the pandemic compared to community-dwelling adults, because these facilities were particularly affected by the COVID-19 pandemic, leading to staffing shortage, visitor restrictions, and new initiatives to bring acute care to these residents in an effort to reduce inter-facility transfers. Fourth, we draw the reader's attention to the fact that we have conducted a sequence of 39 weekly statistical comparisons, and our findings should be interpreted in this context. Finally, the external generalizability of our findings to jurisdictions without universal healthcare access is limited, especially because the pandemic has affected employment, which could negatively impact private health insurance access.

## Conclusion

After an initial reduction in visits for stroke and myocardial infarction, there was a compensatory increase in visits for myocardial infarction. Mortality after ischemic stroke was higher during the lockdown phase than during the other phases. Our findings suggest that people may have been forgoing necessary care during phases of pandemic-related restrictions. Ongoing public health messaging about the urgency of stroke and myocardial infarction and of monitoring care and outcomes is needed.

## Appendix Supplementary materials

Image, application 1

## Footnotes

To access the supplementary material accompanying this article, visit *CJC Open* at https://www.cjcopen.ca/ and at doi:10.1016/j.cjco.2021.06.002.
