# Supplementary material for: Emergency Department Visits, Care, and Outcome After Stroke and Myocardial Infarction During the COVID-19 Pandemic Phases
Source: CJC Open. 2021 Oct 27;3(10):1230–7. doi: 10.1016/j.cjco.2021.06.002 (PMC8548659; doi:10.1016/j.cjco.2021.06.002)

Supplemental Table S1. Administrative health data sources and outcome definitions

Variable	Database	Details
**Age**	RPDB, NACRS	First ED registration date minus date of birth
**Sex**	RPDB, NACRS	ED record linked to RPDB
**Neighborhood income quintile**	Postal Code Conversion File, Census	Using the smallest standard geographic area for which census data are disseminated (400-700 persons) to identify a patient's neighbourhood of residence and the neighbourhood's average income
**Home rural location**	Postal Code Conversion File, Census	Using the smallest standard geographic area for which census data are disseminated (400-700 persons) to determine the extent of urbanicity of a patient's residence<br>Community size = 1 or 2 or 3 Large urban 100+k'<br>Community size = 4 'Medium urban 10-100k'<br>Community size = 5 or 9 'Small town <10k'
**Arrival by ambulance**	NACRS	Arrived at first ED by ambulance
**Evaluation at a regional stroke center**	NACRS	Received care at regional stroke centre at any time during the episode of care
**Intravenous thrombolysis**	NACRS<br>DAD	Patients who received intravenous thrombolysis at any time during the episode of care. Reported in Special Project 340 as yes/no variable (Ref 13 in main paper)
**Endovascular thrombectomy**	DAD	Patients who were treated with EVT at any time during the episode of care. Reported in Special Project 440 as yes/no variable (Ref 14 in main paper)
**Coronary angiogram**	DAD	CCI code: 3IP10 at any time during the episode of care. (Ref 15 in main paper)
**Percutaneous coronary intervention**	DAD	CCI code: 1IJ50, 1IJ54, 1IJ57GQ at any time during the episode of care. (Ref 15 in main paper)
**Coronary artery bypass grafting surgery**	DAD	CCI code: 1IJ76 at any time during the episode of care. (Ref 15 in main paper)
**Death**	RPDB<br>NACRS	Death date (RPDB) – ED registration date (NACRS) <= 30 days
**Admission to hospital**	NACRS	Patient was admitted from ED to inpatient care either at the same facility or as a transfer to another acute care facility

CCI: Canadian Classification of Health Interventions, DAD: Discharge Abstract Database, ED: Emergency Department, EVT: endovascular thrombectomy, NACRS: National Ambulatory Care Reporting System, RPDB: Registered Persons Database

Supplemental Figure S1. Standardized rates of emergency department visits for vascular events per 100,000 people from 2015 to 2020

A) Ischemic stroke or intracerebral hemorrhage

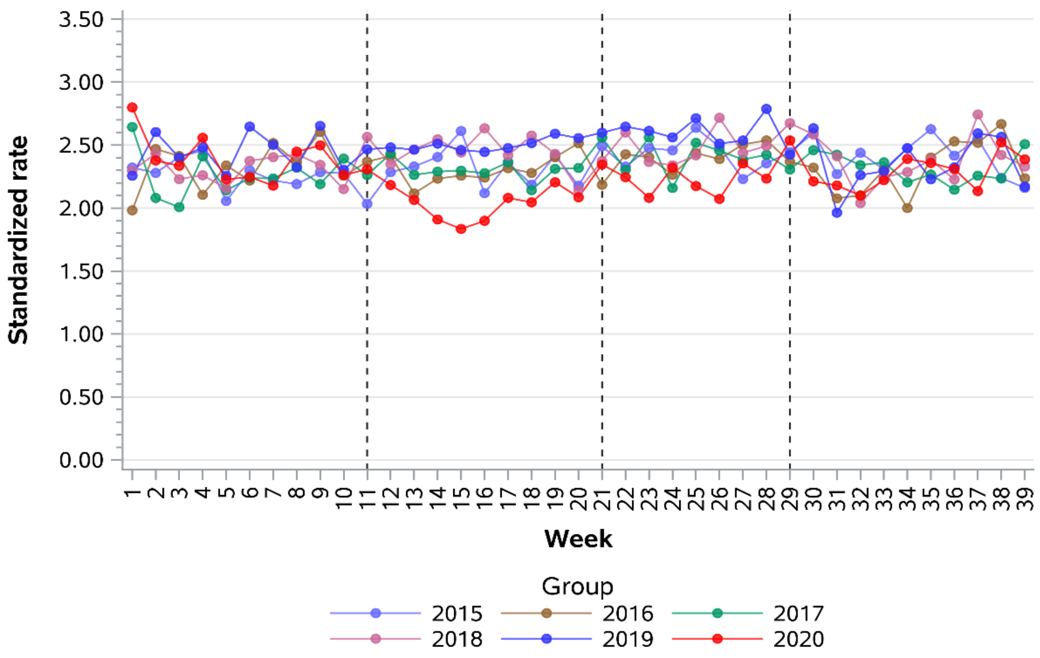

B) Myocardial infarction

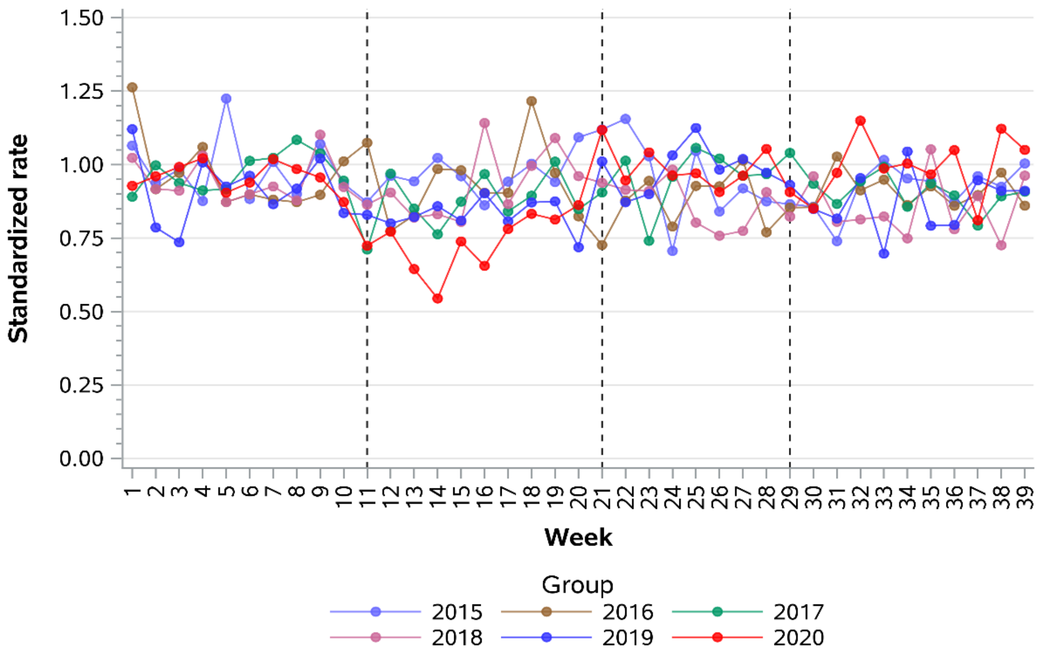

Supplement: Supplementary file 1 [file mmc1.pdf]
